# Preparation of Neohesperidin–Taro Starch Complex as a Novel Approach to Modulate the Physicochemical Properties, Structure and In Vitro Digestibility

**DOI:** 10.3390/molecules28093901

**Published:** 2023-05-05

**Authors:** Youming Zuo, Zirui He, Weidong Yang, Chongde Sun, Xingqian Ye, Jinhu Tian, Xiangli Kong

**Affiliations:** 1 Institute of Nuclear Agricultural Sciences, College of Agriculture and Biotechnology, Zhejiang University, Hangzhou 310058, China; 2 Institute of Fruit Science, College of Agriculture and Biotechnology, Zhejiang University, Hangzhou 310058, China; 3 Institute of Food Processing Engineering, College of Biosystems Engineering and Food Science, Zhejiang University, Hangzhou 310058, China

**Keywords:** neohesperidin, taro starch, physicochemical, structure, digestibility

## Abstract

Neohesperidin (NH), a natural flavonoid, exerts multiple actions, such as antioxidant, antiviral, antiallergic, vasoprotective, anticarcinogenic and anti-inflammatory effects, as well as inhibition of tumor progression. In this study, the NH–taro starch complex is prepared, and the effects of NH complexation on the physicochemical properties, structure and in vitro digestibility of taro starch (TS) are investigated. Results showed that NH complexation significantly affected starch gelatinization temperatures and reduced its enthalpy value (ΔH). The addition of NH increased the viscosity and thickening of taro starch, facilitating shearing and thinning. NH binds to TS via hydrogen bonds and promotes the formation of certain crystalline regions in taro starch. SEM images revealed that the surface of NH–TS complexes became looser with the increasing addition of NH. The digestibility results demonstrated that the increase in NH (from 0.1% to 1.1%, weight based on starch) could raise RS (resistant starch) from 21.66% to 27.75% and reduce RDS (rapidly digestible starch) from 33.51% to 26.76% in taro starch. Our work provided a theoretical reference for the NH–taro starch complex’s modification of physicochemical properties and in vitro digestibility with potential in food and non-food applications.

## 1. Introduction

According to the International Diabetes Federation (IDF), approximately 537 million adults are suffering from diabetes, and this number is predicted to rise to 783 million by 2045. Natural compounds, such as polyphenols, are considered starch-modifying agents, which can potentially produce modified starches without the use of any undesirable chemicals [1,2,3]. Plant polyphenols (mainly including flavonoids and phenolic acids), containing one or multiple hydroxyl groups, are important secondary metabolites; due to their special functionalities such as antioxidant and anti-tumor properties, plant polyphenols are important dietary components beneficial to human health [4,5]. In the last decade, the production of complexes using different polyphenols and starches from various plant sources has been widely reported with the aim of producing functional foods to prevent some public health problems worldwide, such as obesity and diabetes. Polyphenol–starch interactions influence not only the physiochemical properties (including gelatinization, retrogradation and rheological properties) of starch but also, importantly, its digestibility. Compared to native starches, lower digestion rates have been reported for polyphenol–starch complexes, such as pigment–rice starch [6], chlorogenic acid–lotus seed starch [7] and caffeic acid–waxy maize starch [8]. Some polyphenols suppress starch digestion by inhibiting the activities of α-amylase and α-glucosidase, and examples include quercetin [9], proanthocyanidins [10], anthocyanin [11], caffeic acid [8] and ferulic acid [4]. Some polyphenols, for example, proanthocyanidin [12], tea products [13] and tea polyphenols [14], retarded the gelatinization of starches from rice, wheat and potato. Polyphenols, such as quercetin [15] and proanthocyanidin [7,12], have been observed to increase the viscosity of starch and also alter the rheological properties of starches from pregelatinized plasma-modified Tartary buckwheat and proso millet. The surface morphology and crystal structure of starch have been observed to be changed in the presence of polyphenols. Proanthocyanidin–starch pastes of rice, pea and potato displayed a looser and more stretched microstructure [12], and proanthocyanidins reduced the ordered degree of proso millet starch [7] in addition to destroying the long-ordered structure of rice starch [16]. In addition, the maize starch granules were also disintegrated by the addition of increasing quercetin in maize starch [17].

During co-gelatinization, one of the mechanisms by which polyphenols and starches interact is via the hydroxyl and carbonyl groups in polyphenols, which can interact with starches through Van der Waals forces and hydrogen bonds between their hydroxyl and carbonyl groups, which interferes with the reassociation of starch chains; another is that the inner hydrophobic helix of the amylose can accommodate polyphenols stabilized by hydrophobic interactions, which results in the formation of a V-type inclusion complex [13]. Therefore, polyphenols–starch complexes are not chemically modified, and interactions between polyphenols and starches can be applied in starch processing [1,16].

In recent years, due to their lack of apparent toxicity, citrus flavonoids (a type of polyphenol) have attracted wide attention in the food industry as natural food additives [18]. Neohesperidin (NH), which is a citrus flavonoid, is a flavanone glycoside that is mainly present in citrus fruits, especially in the peel, and has important health and industrial benefits [19,20]. NH exerts multiple actions resulting in antioxidant, antiviral, anti-allergic, vasoprotective, anti-carcinogenic and anti-inflammatory effects and inhibition of tumor progression [18,19,21,22,23,24]. NH is shown to be an inhibitor of α-amylase and α-glucosidase and can also interact with starch, slowing the digestion rate of its component amylose as catalyzed by α-amylase and thereby regulating glucose metabolism in vivo [25,26]. However, there are few reports on the effects of NH on starch functional properties. Furthermore, it remains unclear whether NH can inhibit starch digestion under the co-catalysis of both α-amylase and α-glucosidase. The characterization of physicochemical properties is essential to better utilize them in food and non-food applications. However, the effect of NH on the structure and physicochemical properties of starch has not been comprehensively discussed. The feasibility of employing NH as a dietary polyphenol in the starch-based food industry is still unknown.

Taro (*Colocasia esculenta* (L.) Schott), the fifth largest tuber crop in the world, is widely grown in tropical and subtropical regions [27,28]. The content of starch in taro can reach up to 70–80% on a dry weight basis [29]. Taro starch (TS) has irregular, polygonal shapes and small granular sizes (1–5 µm) [30]. Due to its tiny granule size, taro starch can be used as a filler in biodegradable packaging sheets, as a fat substitute and to encapsulate functional ingredients or flavors [31,32]. Due to its high swelling capacity, low amylose content, excellent water and oil absorption capacity and other functional characteristics, taro starch has a huge potential in food processing [33]. Taro starch is easily digestible, which is attributed to its small granule size [34,35]. Excessive intake of taro starch may lead to elevated blood glucose in the human body, and long-term consumption of high-glycemic food can disrupt the blood glucose balance, leading to chronic diseases such as obesity, fatty liver, hyperglycemia and type II diabetes [36]. Based on digestive characteristics, starch can be classified as rapidly digestible starch (RDS), slowly digestible starch (SDS) and resistant starch (RS). RS has many beneficial physiological effects in reducing some diseases, including obesity and diabetes [8,37,38]. Modification of taro starch to produce RS has been widely carried out employing physical, chemical and enzymatic methods either alone or in combination [27].

In this current study, the thermal, rheological properties and the crystal structure of NH–TS complexes were characterized to clarify the physicochemical properties and the structure, and the digestion properties were determined by simulating gastrointestinal digestion. These investigations aimed to explore how neohesperidin complexes with starch and the effects on the functional properties of TS in the resulting NH–TS. Furthermore, our research sheds a light and provides an effective reference for NH as a potential food additive in starch-based food to improve its properties, reduce its digestibility and maintain blood glucose balance in the human body.

## 2. Results and Discussion

### 2.1. Binding Ability of NH with Taro Starch

The binding amount and loading efficiency of NH ranged from 0.067 to 1.439 mg/g and from 6.52% to 15.74%, respectively (Table 1). The binding amount significantly increased continuously when the ratio of NH was increased from 0.1% to 1.1%. The loading efficiency showed an upward trend as more NH was added, and the complex of TS–0.7% NH showed the highest loading efficiency; the results are generally in agreement with a previous report for wheat starch–tannic acid complexes [39]. The binding ability of NH with TS could be affected by the temperature in the process of the starch complexes preparation [40], and the solubility of NH could influence complex formation. Moreover, the increase in hydroxyl groups is expected to enhance steric hindrance between molecules, which may inhibit the complexing of starch and polyphenols [40]. It may be necessary to explore effective methods to improve the efficiency of NH and TS recombination. Some physical approaches have been used to improve the binding of ligands to starch. For example, high-pressure homogenization is a potential strategy to promote the formation of corn starch–fatty acid complexes [41]. Low-power density ultrasound was found to be beneficial to the formation of the amylose–lipid complexes [42]. Co-jet cooking is a rapid consecutive method for the preparation of novel starch-based products and derivatives [43]. Therefore, the exploration of feasible approaches is needed to improve the efficiency of NH and TS recombination in future work.

### 2.2. SEM Images

**Figure 1:**
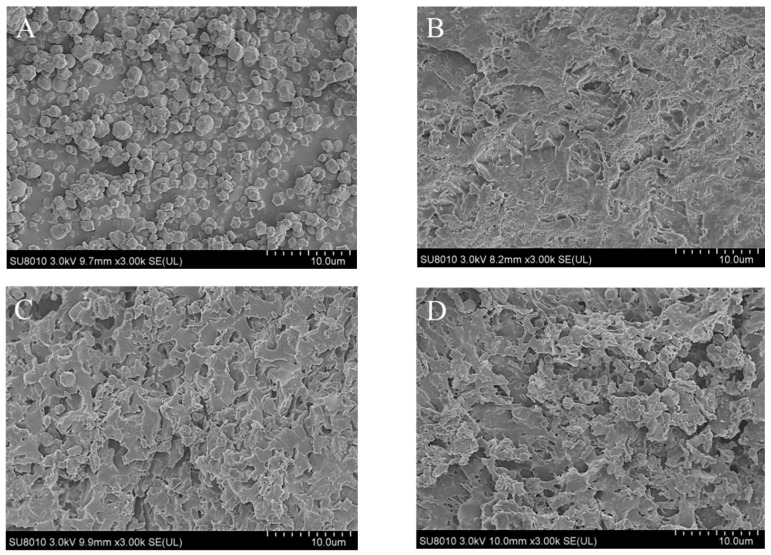
SEM images of native taro starch (**A**), TS with 0.0% NH (**B**), TS with 0.5% NH (**C**) and TS with 1.1% NH (**D**).

Cold field scanning electron microscopy diagrams can intuitively present the effects of polyphenols on the surface morphology of starch granules [5,7,44]. Figure 1 shows SEM images of native TS and NH–TS complexes. Taro starch granules appear small, irregular, polyhedral and nearly spherical with a wrinkled and cracked surface (Figure 1A). Compared with the native TS, gelatinized starch granules appear swollen and broken resulting from the high-temperature treatment [45]. The starch paste condensed together to form an irregular tabular structure with inconspicuous mesh (Figure 1B), and these irregular structures were observed to become more pronounced with increasing ratios of NH. The degree of density changes, and the surface of the complexes observed under electron microscopy became looser with the addition of NH (Figure 1B–D). This result is consistent with a previous work [46], which reported that starch complexed with dandelion flavonoids became less continuous and compact under SEM observation.

Phenolic compounds with many hydroxyl groups attached can form hydrogen bonds with starch molecules, which prevents hydrogen bonds from forming between the starch molecules themselves, resulting in the looser structure of the starch pastes [47]; furthermore, the aggregation of starch–polyphenol complexation and cross-linking allows starch to more easily form a stable and continuous phase after gelatinization [15].

### 2.3. XRD Analysis

**Figure 2:**
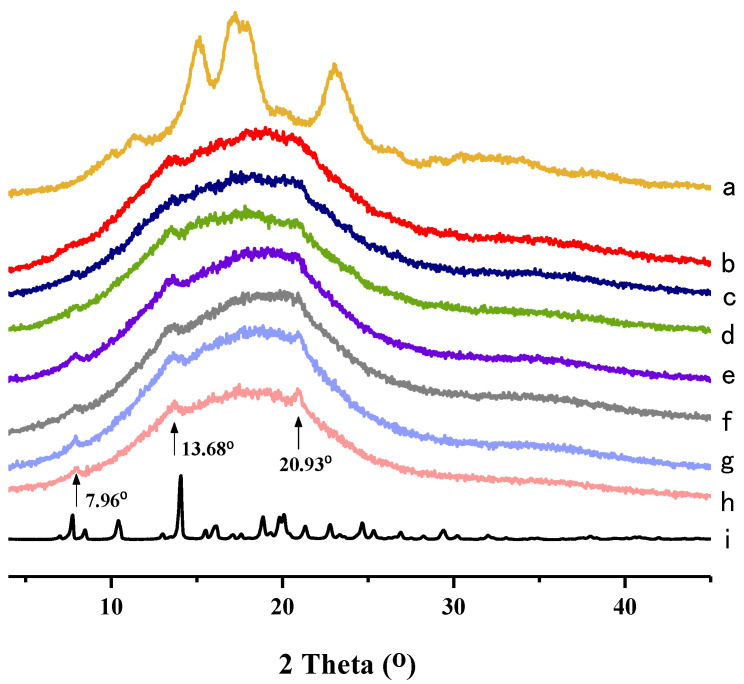
XRD patterns of (**a**) Native TS; (**b**) TS + 0.0% NH; (**c**) TS + 0.1% NH; (**d**) TS + 0.3% NH; (**e**) TS + 0.5% NH; (**f**) TS + 0.7% NH; (**g**) TS + 0.9% NH; (**h**) TS + 1.1% NH; (**i**) NH.

The XRD methodology has been used to observe the long-range ordered structure of starch molecular aggregates, which can reflect the changes in the crystalline and amorphous regions in starch [48]. The crystalline structure of starch can be classified into four types, including A-, B-, C- and V-types [49]. As shown in Figure 2, 4 strong diffraction peaks are observed in native taro starch at 15.1°, 17.2°, 18.0° and 22.9°, which indicates that taro starch has a typical A-type crystallinity [27]. NH showed strong crystal diffraction peaks, implying that NH has a highly crystalline structure [50]. Compared with native taro starch, the gelatinized NH–TS complexes have no obvious peaks characteristic of crystalline structure, suggesting that it has been destroyed by the heating process [51], resulting in the disintegration of the crystalline arrangements of the starch granule helices and partial disruption of the intra- and intermolecular hydrogen bonds between the starch molecules [52].

However, three weak peaks were recorded at 7.96°, 13.68° and 20.93°, which may be due to the formation of new crystalline regions in gelatinized starch and NH–TS complexes (Figure 2b–h). Moreover, at both 7.96° and 20.93°, the peak signals increased in intensity with the additions of NH, which further supports the occurrence of interactions between NH and taro starch. Our findings are in agreement with a previous report on the interaction between caffeic acid and maize starch, where some peaks were present at 28.5 and 32.5° (2θ) with the addition of caffeic acid, which was attributed to formation of a new crystal structure resulting from interactions between maize starch and caffeic acid [4].

### 2.4. FT-IR Analysis

**Figure 3:**
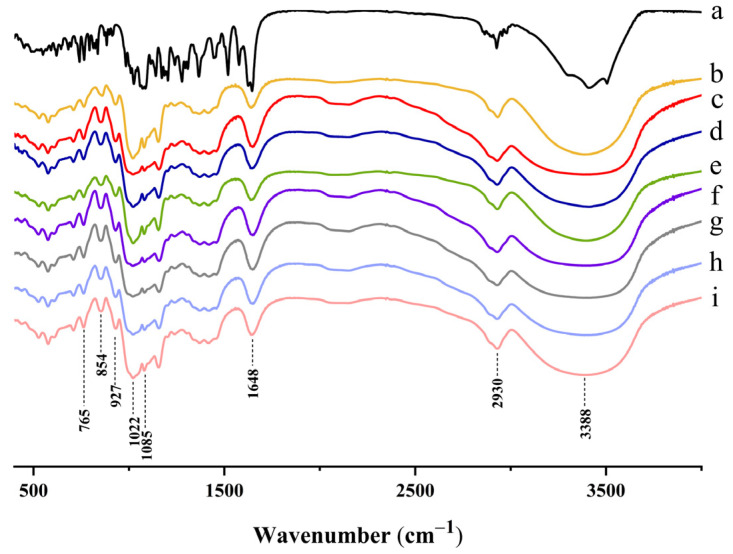
FT-IR spectra of (**a**) NH; (**b**) Native TS; (**c**) TS + 0.0% NH; (**d**) TS + 0.1% NH; (**e**) TS + 0.3% NH; (**f**) TS + 0.5% NH; (**g**) TS + 0.7% NH; (**h**) TS + 0.9% NH; (**i**) TS + 1.1% NH.

FT-IR has been widely used to evaluate the appearance, type and strength of hydrogen bonds, which reflect the changes in starch short range order molecular structure [14]. The infrared absorption peaks and structural features in the infrared spectrogram of these structures (Figure 3) were 3388 cm^−1^ (O–H); 2930 cm^−1^ (C–H); 1648 cm^−1^ (H_2_O); 1085 cm^−1^ (C–O connected to the secondary alcohol hydroxyl group); 1022 cm^−1^ (C–O connected to the primary alcohol hydroxyl group); 927 cm^−1^ (the 1-type absorption band of D-pyran glucose); 854 cm^−1^ (the 2α-type absorption band of D-pyran glucose); and 765 cm^−1^ ( the type 3 absorption band of D-pyran glucose) [53]. Compared to native taro starch, no novel chemical bonds, functional groups or loss of absorption peak were observed in the FT-IR spectra of taro starch when complexed with NH, indicating that there were no chemical bond modifications or covalent bond formation. Significantly, the stretching vibration absorption of the hydrogen bonds from 3000 to 3700 cm^−1^ had a wider range with the increase in NH (Figure 3c–h), which indicates that stronger hydrogen bonds were formed as more NH was added.

It has been shown that the addition of flavonoids with multi-hydroxyls to starch can promote the attachment of flavonoid molecules to the surface of amyloid chains via hydrogen bonding and thus increase the intensity of absorption peaks for O–H [54]. In this study, NH with abundant highly reactive hydroxyl groups could interact with taro starch through hydrogen bonding and compete with intramolecular hydroxyl groups of starch.

### 2.5. Thermal Properties

The gelatinization of starch is a physical transition process. During this process, the amylopectin double helices disassociate, and the crystalline structure is transformed to amorphous. The amount of energy required for the disintegration of double helices is defined as the change in enthalpy [55]. The thermal properties of the native TS and NH–TS complexes are presented in Table 2. The values of T_o_, T_p_ and T_c_ showed an upward trend, from 46.90 to 54.92 °C, from 60.04 to 63.46 °C and from 69.09 to 69.40 °C, respectively, with the increase in ratio of the NH addition to taro starch. These results suggested that polyphenols increased the thermal stability of starch. Similar to our results, previous studies reported that the addition of caffeic acid increased the T_o_, T_p_ and T_c_ values of maize starch [4], and the addition of proanthocyanidin increased the gelatinization temperatures (T_o_, T_p_ and T_c_) of rice starch and potato starch [12]. However, some researchers found that the addition of 10% and 20% longan seed polyphenols did not significantly affect the gelatinization temperatures of starch (T_o_, T_p_ and T_c_). On the contrary, polyphenols reduce the thermal stability of starch [55]. The formed ferulic acid/gallic acid–rice starch complexes had significantly decreased gelatinization temperatures (T_o_, T_p_ and T_c_) [5]; the presence of proanthocyanidins decreased the T_o_, T_p_ and T_c_ values of the proso millet starch [48]; and proanthocyanidins addition decreased the gelatinization temperatures (T_o_, T_p_ and T_c_) of potato starch [12]. Therefore, the different influences of polyphenol–starch interactions on gelatinization parameters might be dependent on both types of polyphenols and molecular structure of starches.

Gelatinization enthalpy depicts the energy required for destroying the double-helix structure and eliminating crystallinity within the starch granules, which is an indicator of the loss of molecular order structure based on interactions between the particles [12,14,16,56]. As shown in Table 2, the value of gelatinization enthalpy (ΔH) displayed a remarkable decrease trend from 5.73 to 4.52 J/g with NH addition increasing, indicating that the ordered structure of taro starch was almost destroyed, and the starch crystallinity decreased [16,52]. Similar results have been widely reported in previously prepared polyphenol–starch complexes, such as longan seed polyphenols–maize starch [55], tea polyphenols–starch [14], caffeic acid–maize starch [4] and proanthocyanidin–proso millet starch complexes [48]. Starch with lower ΔH can more readily gelatinize, and less energy is required to disrupt double helices [14]. A high value of ΔH suggests that more energy is needed to unwind and break bonds during the process of gelatinization due to the tight structure of the gelatinized starch particles. This is supported by the observations in SEM images, as shown in Figure 1B–D.

### 2.6. Flow Behaviors

**Figure 4:**
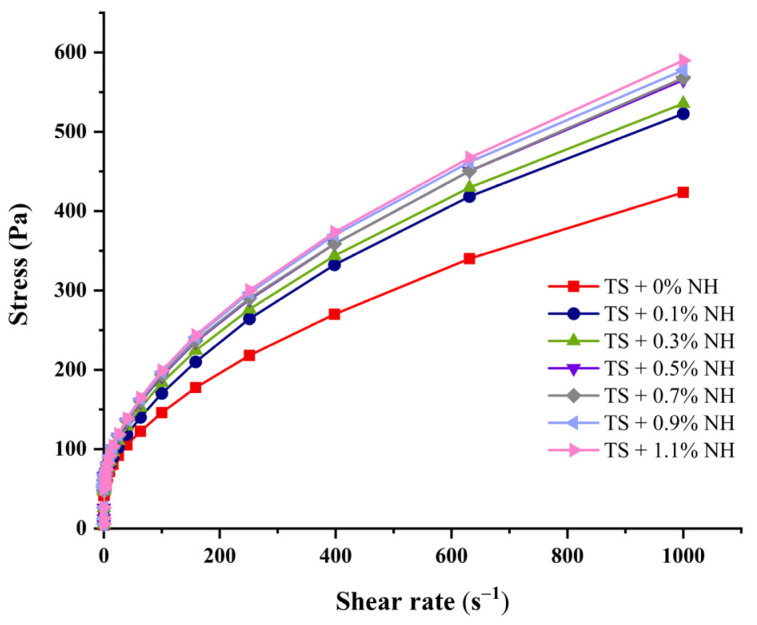
Rheological curves of gelatinized starch with different ratios of NH.

The steady rheological curves of the TS–NH complexes are shown in Figure 4. The shear stress required for starch pastes during the flow process of the complexes became stronger with the increase in shear rate and addition of NH (Figure 4). The flow properties of complexes are positively correlated with the NH ratios.

The flow parameters based on the Herschel–Bulkley model are presented in Table 3. The correlation coefficients (R^2^) of all curves were above 0.99 (Table 3), which indicates the good fit achieved for the rheological behaviors of our samples based on the Herschel–Bulkley model. The value of *n* reflects the proximity of the system behavior to that of a Newtonian fluid, when *n* is equal to 1 and the system behavior is a Newtonian fluid. Otherwise, the behavior is pseudoplastic when *n* is lower than 1 [57]. The values of *σ* (yield stress > 0) and *n* (rate index) < 1 further indicate that the pastes formed by native taro starch and TS–NH complexes are typical pseudoplastic fluids and show shear-thinning behavior [58]. Since the n value decreases with the increase in NH, it could be seen that the addition of NH results in starch pastes more readily shearing and thinning. Consistency is a function of viscosity, and higher consistency coefficients (*K*) values indicate higher viscosity [59]. The values of *K* and viscosity show a significant upward trend (Table 3), indicating that NH can promote starch thickening and pseudoelasticity. In previous reports, it was shown that lotus leaf flavonoids can increase the consistency coefficient of soluble starch [59], and quercetin increases the apparent viscosity of Tartary buckwheat starch [44]. The increased consistency of the starch–polyphenol complexes might be attributed to the strong and continuous network that is formed. The interaction between polyphenols and starch via hydrogen bonding enhances the entanglement of starch chains and the system viscosity, which might result in the observed phenomena [15,44].

### 2.7. In Vitro Digestibility

**Figure 5:**
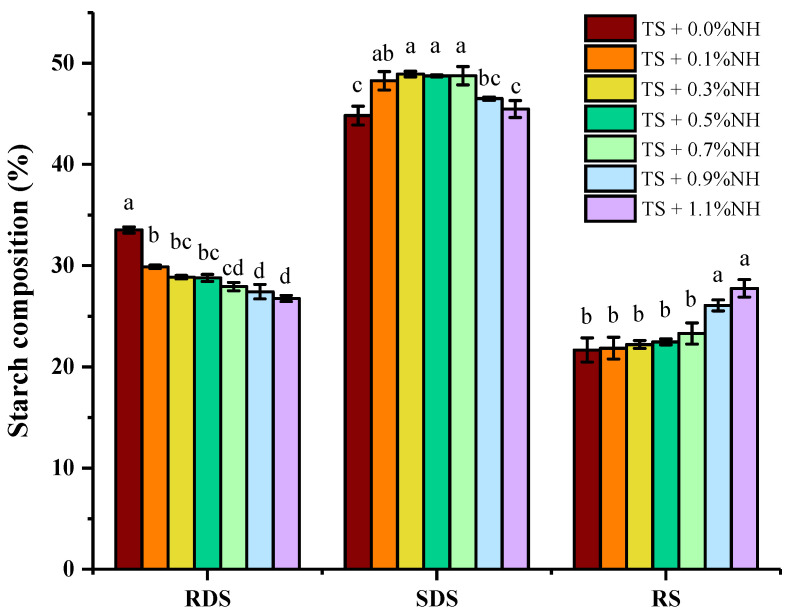
Contents of RDS, SDS and RS fractions in different TS–NH complexes. Bars with different letters within the same group indicate significant differences (*p <* 0.05).

Starch nutritional compositions (RDS, SDS and RS) of the samples are depicted in Figure 5. Gelatinized starch is composed of 33.51% RDS, 44.82% SDS and 21.66% RS, respectively. As shown in Figure 5, the percentage of RDS decreased from 33.51% to 26.76%, and the proportion of RS showed an upward trend from 21.66% to 27.75% with an increase in the percentage of NH from 0.0% to 1.1%. These findings confirmed that NH can indeed interact with taro starch and inhibit its rate of digestion, which, in vivo, might contribute to lowering the blood sugar balance in the body after meals. These results were in agreement with those of previous studies, which found that flavonoid–starch complexes, such as quercetin/rutin–Tartary buckwheat starch and kaempferol–rice starch, exhibit anti-digestive effects [9,60,61].

At the first stage of digestive reaction, α-amylase can randomly and indiscriminately excise on α-1,4 glycoside bonds in the dextran molecules and produce glucose, maltose and extreme dextrin [62]; then, amyloglucosidase (an exonuclease) sequentially cuts glucose from the non-reducing end of the starch chain, resulting in a single end-product of glucose [63]. Therefore, we believe that α-amylase plays a vital role in the digestive process. Polyphenol–starch complexes have been considered poor substrates for α-amylase [46]. Some previous reports have suggested that flavonoids can competitively inhibit amylase activity or suppress the activity of enzymes directly and, thus, reduce the rate of starch digestion [9,44,64]. In the current work, we could draw a conclusion that the binding of NH to TS may result in steric hindrance, which would reduce the adhesion of amylase to the starch paste, ultimately affecting the rate of starch hydrolysis. In another aspect, the enzymatic hydrolysis of the starch is accompanied by the release of polyphenols [7,16], so liberated and dispersed NH may bind enzymes and inhibit the activities, a possibility that needs to be verified by further study. Additionally, the optimal ratio of NH for delaying starch digestion was not characterized in the current work. Future research is recommended to determine the optimal ratio between NH and starch by increasing the amount of NH addition to achieve the highest possible inhibition of starch digestion for regulating glucose metabolism in vivo.

## 3. Materials and Methods

### 3.1. Materials

Taro was sourced from Fenghua Research Institute, NingBo University of Technology, and taro starch was extracted according to the method described in our previous report after first cutting the taro tubers into small pieces [65]. NH (CAS:13241-33-3, purity ≥ 95%) was purchased from Shanghai Xushuo Biotech Co., Ltd. (Shanghai, China). The glucose assay kits were purchased from Ningbo Saike Biotech Co., Ltd. (Ningbo, China). Pancreatin from porcine pancreas (CAS:8049476, α-amylase activity ≥ 100 U/mg) and amyloglucosidase (CAS:9032-08-0, 105 U/mL) were purchased from Aladdin Co., Ltd. (Shanghai, China). All other chemicals in this study were analytical grade reagents.

### 3.2. Preparation of NH–Taro Starch Complexes

NH–taro starch complexes were prepared as per Zheng et al. with some modifications [16]. Taro starches (10 g, dry basis) and different ratios of NH (0%, 0.1%, 0.3%, 0.5%, 0.7%, 0.9%, 1.1%, *w*/*w*) were combined and dispersed in 100 mL 40% ethanol and boiled for 20 min with continuous stirring until completely gelatinized. The suspensions were cooled down to room temperature and then centrifuged at 5000 rpm for 15 min. The sediments were washed with 40% ethanol twice, and then some of them were used for rheological analysis and digestibility experiments, and the remainder were lyophilized and crushed into powder (through a 100 mesh) for other routine analyses. All supernatants collected after washing were further used for analysis of binding ability by HPLC.

### 3.3. Ability of NH to Bind Taro Starch

The concentrations of NH in the supernatants and washed solutions were analyzed according to a previous report [26]. Briefly, concentrations of NH were determined using a Waters e2695 Analytical High Performance Liquid Chromatography system with a 2998 Diode Array Detector and an ODS C18 analytical column (4.6 × 250 mm) (Waters Co., USA). Milli-Q water (A) and acetonitrile (B) at a ratio of 79:21 (*v*/*v*) were used as the mobile phase of HPLC at a velocity of 1 mL/min. The detection wavelength was 280 nm, and the column temperature was set to 30 °C. The amount of NH was determined based on the retention time and the chromatographic peak area. The concentrations of NH were obtained based on the standard curve. The binding amount and loading efficiency were calculated with the following equations [10]:

Binding amount (mg/g) = (W_1_ − W_2_)/W_3_

Loading efficiency (%) = (W_1_ − W_2_)/W_1_

where W_1_, W_2_ and W_3_ represent the weight of initial NH, free NH and taro starch, respectively.

### 3.4. Scanning Electron Microscope (SEM)

The microstructure of the gelatinized starch was observed using a scanning electron microscope (TM-1000, Hitachi, Japan). The starch powder was dispersed and uniformly placed on the carrier platform before conductive coating in a sputter coater (SCD 050, Balzers, Liechtenstein) was performed, and then the sample was then firmly fixed by a rubber ear syringe. The images were taken under an acceleration voltage of 2 kV and were photographed at 3000× magnification according to a previous report [66].

### 3.5. X-ray Diffraction (XRD)

X-ray diffraction patterns were analyzed using a Siemens D5005 X-ray diffractometer (Bruker AXS, Karlsruhe, Germany) equipped with a copper tube, producing Cu-Kα (1.54 Å) radiation. The diffractograms were obtained by scanning at the angle ranging from 4° to 45° at a rate of 1°/min (2θ) and a step size of 0.02° (2θ), and the accelerated voltage and current were set to 40 kV and 40 mA [59].

### 3.6. Fourier Transform Infrared Spectroscopy (FT-IR)

The ordered structure of the complexes of NH and taro starch was investigated using a Thermo Scientific Nicolet IS50 spectrometer equipped with an attenuated total reflectance (ATR) sampling accessory (Thermo Fisher Scientific Co., Waltham, MA, USA). The powder samples (2 mg) were pressed into KBr pellets (200 mg), and Milli-Q water was used as the blank control. Spectra were automatically baseline-corrected by OMNIC 8.0 software and deconvoluted over the range from 4000 to 400 cm^−1^ [46].

### 3.7. Differential Scanning Calorimetry (DSC)

The gelatinization properties of NH–TS complexes were determined by using a differential scanning calorimeter (Q100, TA Instruments, New Castle, DE, USA) following our previous method [67]. The samples (about 2 mg) were weighed and placed in an aluminum pan, and 6 μL ddH_2_O was then added. The samples in DSC pans for gelatinization were equilibrated at 4 °C for 48 h before testing. The samples were measured during run heating from 25 to 95 °C at a rate of 10 °C/min under 20 mL/min flowing nitrogen, and an empty sealed pan was used as a reference. The onset temperature (T_o_), peak temperature (T_p_), conclusion temperature (T_c_) and enthalpy of melting (ΔH) were calculated from the DSC endothermic curves.

### 3.8. Rheological Properties

Static rheological properties of taro starch with different ratios of NH were determined using a Discovery Hybrid Rheometer (DHR-1, TA Instruments, USA) equipped with a parallel plate geometry [58]. NH–starch complexes of 10% (*w*/*v*) in distilled water were prepared using screw cap centrifuge tubes. The shear rates were increased from 0.1 to 1000 s^−1^ at 25 °C. The Herschel–Bulkley equation was used to fit the experimental data in regression as follows:

*σ* = *σ_0_* + *Kγ^n^*

where *σ*, *σ_0_*, *K*, *γ* and n represent shear stress (Pa), yield stress (Pa), consistency coefficient (Pa·s^n^), shear rate (s^−1^) and rate index (dimensionless), respectively.

### 3.9. In Vitro Digestion

Enzyme hydrolysis of NH–TS complexes was performed according to a previous method [68]. The gelatinized samples mixed with sodium acetate buffer (0.1 M, pH 5.2, plus 1 M CaCl_2_) were incubated at 37 °C together with the enzyme solutions (18 mg pancreatin, 12 µL amyloglucosidase). Then, 1 mL mixed enzyme solution was added to the samples while being constantly vibrated in a 37 °C water bath. The hydrolysates (1 mL) were sampled at 20 min and 120 min by transferring into 20 mL 80% ethanol (to inactivate the enzymes) and then centrifuged at 4000 rpm for 10 min. The glucose content from the supernatant was determined using a glucose assay kit described in Section 2.1. The starch constitutes were calculated as follows:

RDS = G_20_ × 0.9

SDS = (G_120_ − G_20_) × 0.9

RS = TTS − (G_120_ × 0.9)

where RDS, SDS, RS and TTS represent rapidly digestible starch, slowly digestible starch, resistant starch and total taro starch, respectively [69]. G_20_ and G_120_ are the glucose contents of the digestive solutions at 20 min and 120 min, respectively; 0.9 is the conversion factor from starch to glucose.

### 3.10. Statistical Analysis

All experiments were performed at least three times. All data were analyzed by one-way analysis of variance (ANOVA) using SPSS 19 statistical software, and Duncan’s test was used to compare the means at a significance level of *p* < 0.05.

## 4. Conclusions

The amount of binding between NH and TS increased with the ratio of NH. NH significantly influences the gelatinization parameters of TS, and NH enhances the thermal stability and decreases the enthalpy value of taro starch in facilitating gelatinization. The viscosity and shear stress of TS–NH complex pastes increased with the ratio of added NH. NH alters the granule morphology and crystal structure of TS remarkably. FT-IR spectra confirmed that the binding modes of TS–NH are based on non-covalent bonding (hydrogen bonding). TS–NH complexes showed lower digestion efficiency and increased the RS fractions in starch. The effects are dependent on the amount of NH added. Our results demonstrated that NH can be employed to improve the health effects and edible value of TS by modifying its quality and provided a theoretical reference with NH–taro starch complex for modification of the physicochemical properties and in vitro digestibility with potential in food and non-food applications.

## Figures and Tables

**Table 1:** The binding content and loading efficiency of NH in NH–TS complexes.

Samples	Binding Amount (mg/g)	Loading Efficiency (%)
TS + 0.1% NH	0.067 ± 0.004 ^f^	6.52 ± 0.36 ^e^
TS + 0.3% NH	0.340 ± 0.018 ^e^	11.33 ± 0.60 ^d^
TS + 0.5% NH	0.664 ± 0.004 ^d^	13.25 ± 0.07 ^bc^
TS + 0.7% NH	1.103 ± 0.008 ^c^	15.74 ± 0.11 ^a^
TS + 0.9% NH	1.229 ± 0.007 ^b^	13.64 ± 0.07 ^b^
TS + 1.1% NH	1.439 ± 0.012 ^a^	13.06 ± 0.11 ^c^

Note: Values (mean ± SD) in the same column with different letters indicate significant differences (*p* < 0.05).

**Table 2:** Thermal properties of gelatinized taro starch with and without NH.

NH (%)	T_o_ (°C)	T_p_ (°C)	T_c_ (°C)	ΔH (J/g)
0	46.90 ± 0.41 ^d^	60.04 ± 0.26 ^c^	69.09 ± 0.95 ^a^	5.73 ± 0.14 ^a^
0.10	51.11 ± 2.24 ^c^	62.15 ± 0.96 ^b^	69.10 ± 0.51 ^a^	5.47 ± 0.29 ^ab^
0.30	52.63 ± 1.71 ^bc^	61.94 ± 0.97 ^b^	69.10 ± 1.16 ^a^	5.43 ± 1.21 ^ab^
0.50	53.45 ± 0.45 ^ab^	63.15 ± 0.20 ^a^	69.29 ± 0.69 ^a^	5.41 ± 0.25 ^ab^
0.70	54.37 ± 0.36 ^ab^	63.44 ± 0.05 ^a^	69.39 ± 0.39 ^a^	5.15 ± 0.35 ^ab^
0.90	54.92 ± 0.41 ^a^	63.46 ± 0.33 ^a^	69.40 ± 0.64 ^a^	4.81 ± 0.17 ^ab^
1.10	55.27 ± 0.71 ^a^	63.43 ± 0.03 ^a^	69.46 ± 1.53 ^a^	4.52 ± 0.27 ^b^

Note: Values (mean ± SD) in the same column with different letters indicate significant differences (*p* < 0.05).

**Table 3:** The flow behavior parameters of the taro starch with and without NH fitted to the Herschel–Bulkley model.

Samples	*σ*_0_ (Pa)	Viscosity (Pa·s)	*K* (Pa·s^n^)	*n*	R^2^
TS + 0% NH	30.199 ± 0.297 ^d^	9.536 ± 0.386 ^d^	8.916 ± 0.561 ^c^	0.552 ± 0.004 ^a^	0.996
TS + 0.1% NH	32.488 ± 0.372 ^c^	10.645 ± 0.273 ^c^	10.925 ± 0.172 ^b^	0.553 ± 0.000 ^a^	0.998
TS + 0.3% NH	37.051 ± 0.309 ^b^	13.558 ± 0.216 ^b^	10.933 ± 0.143 ^b^	0.515 ± 0.007 ^b^	0.994
TS + 0.5% NH	38.741 ± 0.330 ^a^	14.441 ± 0.401 ^a^	11.869 ± 0.233 ^a^	0.513 ± 0.002 ^b^	0.992
TS + 0.7% NH	36.794 ± 0.409 ^b^	14.507 ± 0.353 ^a^	11.909 ± 0.277 ^a^	0.517 ± 0.001 ^b^	0.993
TS + 0.9% NH	38.881 ± 1.545 ^a^	14.623 ± 0.260 ^a^	11.964 ± 0.166 ^a^	0.514 ± 0.000 ^b^	0.994
TS + 1.1% NH	38.820 ± 0.836 ^a^	14.680 ± 0.280 ^a^	12.012 ± 0.133 ^a^	0.504 ± 0.000 ^c^	0.994

Note: Considering the variations among the three measurements were narrow, representative data were selected; values (mean ± SD) in the same column with different letters indicate significant differences (*p* < 0.05).

## Data Availability

The data will be made available upon reasonable request.

## Sample Availability Statement

Samples of the compounds are available from the authors.

## References

[1] Oladele A.K., Duodu K.G., Emmambux N.M. (2019). Pasting, flow, thermal and molecular properties of maize starch modified with crude phenolic extracts from grape pomace and sorghum bran under alkaline conditions. Food Chem..

[2] Iqbal S., Nadeem S., Bano R., Bahadur A., Ahmad Z., Javed M., AL-Anazy M.M., Qasier A.A., Laref A., Shoaib M. (2021). Green synthesis of biodegradable terpolymer modified starch nanocomposite with carbon nanoparticles for food packaging application. J. Appl. Polym. Sci..

[3] Iqbal S., Nadeem S., Bahadur A., Javed M., Ahmad Z., Ahmad M.N., Shoaib M., Liu G.C., Mohyuddin A., Raheel M. (2021). The Effect of Ni-Doped ZnO NPs on the Antibacterial Activity and Degradation Rate of Polyacrylic Acid-Modified Starch Nanocomposite. JOM.

[4] Zheng Y., Tian J., Kong X., Yang W., Yin X., Xu E., Chen S., Liu D., Ye X. (2020). Physicochemical and digestibility characterisation of maize starch–caffeic acid complexes. LWT-Food Sci. Technol..

[5] Han X., Zhang M., Zhang R., Huang L., Jia X., Huang F., Liu L. (2020). Physicochemical interactions between rice starch and different polyphenols and structural characterization of their complexes. LWT-Food Sci. Technol..

[6] Fan M., Lian W., Li Y., Qian H., Zhang H., Rao Z., Wang L. (2022). Evaluation of the physicochemical properties and in vitro digestibility of the complex formed between rice starch and a novel pigment from *Vaccinium bracteatum* Thunb. leaf. Food Chem..

[7] Jiang X., Wang J., Ou Y., Zheng B. (2021). Effect of chlorogenic acid on the structural properties and digestibility of lotus seed starch during microwave gelatinization. Int. J. Biol. Macromol..

[8] Han M., Bao W., Wu Y., Ouyang J. (2020). Insights into the effects of caffeic acid and amylose on in vitro digestibility of maize starch-caffeic acid complex. Int. J. Biol. Macromol..

[9] Zhou Y., Jiang Q., Ma S., Zhou X. (2021). Effect of quercetin on the in vitro Tartary buckwheat starch digestibility. Int. J. Biol. Macromol..

[10] Zhang Z., Tian J., Fang H., Zhang H., Kong X., Wu D., Zheng J., Liu D., Ye X., Chen S. (2020). Physicochemical and Digestion Properties of Potato Starch Were Modified by Complexing with Grape Seed Proanthocyanidins. Molecules.

[11] Ji Y., Liu D., Jin Y., Zhao J., Zhao J., Li H., Li L., Zhang H., Wang H. (2021). In vitro and in vivo inhibitory effect of anthocyanin-rich bilberry extract on α-glucosidase and α-amylase. LWT-Food Sci. Technol..

[12] Xu J., Dai T., Chen J., He X., Shuai X., Liu C., Li T. (2021). Effects of three types of polymeric proanthocyanidins on physicochemical and *in vitro* digestive properties of potato starch. Foods.

[13] Lv Y., Li M., Pan J., Zhang S., Jiang Y., Liu J., Zhu Y., Zhang H. (2020). Interactions between tea products and wheat starch during retrogradation. Food Biosci..

[14] Li H., Zhai F., Li J., Zhu X., Guo Y., Zhao B., Xu B. (2021). Physicochemical properties and structure of modified potato starch granules and their complex with tea polyphenols. Int. J. Biol. Macromol..

[15] Li Y., Gao S., Ji X., Liu H., Liu N., Yang J., Lu M., Han L., Wang M. (2020). Evaluation studies on effects of quercetin with different concentrations on the physicochemical properties and in vitro digestibility of Tartary buckwheat starch. Int. J. Biol. Macromol..

[16] Zheng Y., Tian J., Kong X., Wu D., Chen S., Liu D., Ye X. (2021). Proanthocyanidins from Chinese berry leaves modified the physicochemical properties and digestive characteristic of rice starch. Food Chem..

[17] Zhang L., Yang X., Li S., Gao W. (2011). Preparation, physicochemical characterization and in vitro digestibility on solid complex of maize starches with quercetin. LWT-Food Sci. Technol..

[18] Gong N., Zhang B., Yang D., Gao Z., Du G., Lu Y. (2015). Development of new reference material neohesperidin for quality control of dietary supplements. J. Sci. Food Agric..

[19] Karim N., Shishir M.R.I., Rashwan A.K., Ke H., Chen W. (2021). Suppression of palmitic acid-induced hepatic oxidative injury by neohesperidin-loaded pectin-chitosan decorated nanoliposomes. Int. J. Biol. Macromol..

[20] Wang C., Xia N., Yu M., Zhu S. (2023). Physicochemical properties and mechanism of solubilised neohesperidin system based on inclusion complex of hydroxypropyl-β-cyclodextrin. Int. J. Biol. Macromol..

[21] Tong L., Zhou D., Gao J., Zhu Y., Sun H., Bi K. (2012). Simultaneous determination of naringin, hesperidin, neohesperidin, naringenin and hesperetin of *Fractus aurantii* extract in rat plasma by liquid chromatography tandem mass spectrometry. J. Pharm. Biomed. Anal..

[22] Martati E., Utari D.P., Wulan S.N. (2022). Modeling the influence of extraction parameters on the recovery of antioxidant compounds of microwave extracted citrus (*Citrus reticulata*) peel by Response Surface Methodology. Curr. Anal. Chem..

[23] Huzio N., Grytsyk A., Raal A., Grytsyk L., Koshiovyi O. (2022). Phytochemical and pharmacological research in *Agrimonia eupatoria* L. herb extract with anti-inflammatory and hepatoprotective properties. Plants.

[24] Zeng X., Zheng Y., He Y., Zhang J., Peng W., Su W. (2022). Microbial metabolism of naringin and the impact on antioxidant capacity. Nutrients.

[25] Shen W., Xu Y., Lu Y.H. (2012). Inhibitory Effects of citrus flavonoids on starch digestion and antihyperglycemic effects in HepG2 cells. J. Agric. Food Chem..

[26] Zhang J., Sun C., Yan Y., Chen Q., Luo F., Zhu X., Li X., Chen K. (2012). Purification of naringin and neohesperidin from Huyou (*Citrus changshanensis*) fruit and their effects on glucose consumption in human HepG2 cells. Food Chem..

[27] Nagar C.K., Dash S.K., Rayaguru K., Pal U.S., Nedunchezhiyan M. (2021). Isolation, characterization, modification and uses of taro starch: A review. Int. J. Biol. Macromol..

[28] Zhu X., Cui W., Zhang E., Sheng J., Yu X., Xiong F. (2018). Morphological and physicochemical properties of starches isolated from three taro bulbs. Starch-Stärke.

[29] Kapcum C., Pasada K., Kantiwong P., Sroysang B., Phiwtawee J., Suphantharika M., Belur P.D., Agoo E.M.G., Janairo J.I.B., Wongsagonsup R. (2022). Effects of different cooking methods on chemical compositions, in vitro starch digestibility and antioxidant activity of taro (*Colocasia esculenta*) corms. Int. J. Food Sci. Technol..

[30] Jane J., Shen L., Chen J., Lim S., Kasemsuwan T., Nip W.K. (1992). Physical and chemical studies of taro starches and flours. Cereal Chem..

[31] Lim S.T., Jane J.L., Rajagopalan S., Seib P.A. (1992). Effect of starch granule size on physical properties of starch-filled polyethylene film. Biotechnol. Prog..

[32] Zhao J., Whistler R. (1994). Spherical aggregates of starch granules as flavor carriers. Food Technol..

[33] Singla D., Singh A., Dhull S.B., Kumar P., Malik T., Kumar P. (2020). Taro starch: Isolation, morphology, modification and novel applications concern—A review. Int. J. Biol. Macromol..

[34] Sit N., Deka S.C., Misra S. (2015). Optimization of starch isolation from taro using combination of enzymes and comparison of properties of starches isolated by enzymatic and conventional methods. J. Food Sci. Technol..

[35] Wang X., Reddy C.K., Xu B. (2018). A systematic comparative study on morphological, crystallinity, pasting, thermal and functional characteristics of starches resources utilized in China. Food Chem..

[36] Tian J., Ogawa Y., Shi J., Chen S., Zhang H., Liu D., Ye X. (2019). The microstructure of starchy food modulates its digestibility. Crit. Rev. Food Sci. Nutr..

[37] Simsek S., EI S.N. (2012). Production of resistant starch from taro (*Colocasia esculenta* L. Schott) corm and determination of its effects on health by *in vitro* methods. Carbohydr. Polym..

[38] Naidoo K., Amonsou E.O., Oyeyinka S.A. (2015). *In vitro* digestibility and some physicochemical properties of starch from wild and cultivated amadumbe corms. Carbohydr. Polym..

[39] Kan L.J., Capuano E., Oliviero T., Renzetti S. (2022). Wheat starch-tannic acid complexes modulate physicochemical and rheological properties of wheat starch and its digestibility. Food Hydrocoll..

[40] Le Bourvellec C., Guyot S., Renard C.M.G.C. (2004). Non-covalent interaction between procyanidins and apple cell wall material. Part, I. Effect of some environmental parameters. Biochim. Biophys. Acta-Gen. Subj..

[41] Meng S., Ma Y., Cui J., Sun D.W. (2014). Preparation of corn starch–fatty acid complexes by high-pressure homogenization. Starch-Stärke.

[42] Liu P., Wang R., Kang X., Cui B., Yu B. (2018). Effects of ultrasonic treatment on amylose-lipid complex formation and properties of sweet potato starch-based films. Ultrason. Sonochem..

[43] Fanta G.F., Shogren R.L., Salch J.H. (1999). Steam jet cooking of high-amylose starch–fatty acid mixtures. An investigation of complex formation. Carbohydr. Polym..

[44] Gao S., Liu H., Sun L., Cao J., Yang J., Lu M., Wang M. (2021). Rheological, thermal and in vitro digestibility properties on complex of plasma modified Tartary buckwheat starches with quercetin. Food Hydrocoll..

[45] Lefnaoui S., Moulai-Mostefa N. (2015). Synthesis and evaluation of the structural and physicochemical properties of carboxymethyl pregelatinized starch as a pharmaceutical excipient. Saudi Pharm. J..

[46] Huang Y., Wu P., Chen X. (2022). Mechanistic insights into the influence of flavonoids from dandelion on physicochemical properties and in vitro digestibility of cooked potato starch. Food Hydrocoll..

[47] Yang J.P., He H., Lu Y.H. (2014). Four flavonoid compounds from *Phyllostachys edulis* leaf extract retard the digestion of starch and its working mechanisms. J. Agric. Food Chem..

[48] Xiao Y., Zheng M., Yang S., Li Z., Liu M., Yang X., Lin N., Liu J. (2021). Physicochemical properties and in vitro digestibility of proso millet starch after addition of proanthocyanidins. Int. J. Biol. Macromol..

[49] Zobel H.F. (1988). Starch crystal transformations and their industrial importance. Starch-Stärke.

[50] Xia N., Wang C., Zhu S. (2022). Interaction between pH-shifted ovalbumin and insoluble neohesperidin: Experimental and binding mechanism studies. Food Chem..

[51] Wu Y., Chen Z., Li X., Li M. (2009). Effect of tea polyphenols on the retrogradation of rice starch. Food Res. Int..

[52] Lv Y., Zhang L., Li M., He X., Hao L., Dai Y. (2019). Physicochemical properties and digestibility of potato starch treated by ball milling with tea polyphenols. Int. J. Biol. Macromol..

[53] Shibanuma K., Takeda Y., Hizukuri S., Shibata S. (1994). Molecular structures of some wheat starches. Carbohydr. Polym..

[54] Zhou X., Chen J., Wang S., Zhou Y. (2022). Effect of high hydrostatic pressure treatment on the formation and in vitro digestion of Tartary buckwheat starch/flavonoid complexes. Food Chem..

[55] He T., Wang K., Zhao L., Chen Y., Zhou W., Liu F., Hu Z. (2021). Interaction with longan seed polyphenols affects the structure and digestion properties of maize starch. Carbohydr. Polym..

[56] Li C., Gilbert R.G. (2016). Progress in controlling starch structure by modifying starch-branching enzymes. Planta.

[57] Lu J., Luo Z., Xiao Z. (2012). Effect of lysine and glycine on pasting and rheological properties of maize starch. Food Res. Int..

[58] Kong X., Kasapis S., Bertoft E., Corke H. (2010). Rheological properties of starches from grain amaranth and their relationship to starch structure. Starch-Stärke.

[59] Wang M., Shen Q., Hu L., Hu Y., Ye X., Liu D., Chen J. (2018). Physicochemical properties, structure and *in vitro* digestibility on complex of starch with lotus (*Nelumbo nucifera* Gaertn.) leaf flavonoids. Food Hydrocoll..

[60] Wang L., Wang L., Wang T., Li Z., Gao Y., Cui S.W., Qiu J. (2022). Comparison of quercetin and rutin inhibitory influence on Tartary buckwheat starch digestion in vitro and their differences in binding sites with the digestive enzyme. Food Chem..

[61] Peng L., Wei L., Yi Q., Chen G., Yao Z., Yan Z., Zhao G. (2019). *In vitro* potential of flavonoids from tartary buckwheat on antioxidants activity and starch digestibility. Int. J. Food Sci. Technol..

[62] Janeček Š., Svensson B., MacGregor E.A. (2014). α-Amylase: An enzyme specificity found in various families of glycoside hydrolases. Cell. Mol. Life Sci..

[63] Kelly J.J., Alpers D.H. (1973). Properties of human intestinal glucoamylase. Biochim. Biophys. Acta-Enzymol..

[64] Du J., Yao F., Zhang M., Khalifa I., Li K., Li C. (2019). Effect of persimmon tannin on the physicochemical properties of maize starch with different amylose/amylopectin ratios. Int. J. Biol. Macromol..

[65] Kong X., Bao J., Corke H. (2009). Physical properties of *Amaranthus* starch. Food Chem..

[66] Zheng Y., Tian J., Ogawa Y., Yin X., Xu E., Chen S., Liu D., Kong X., Ye X. (2021). Co-extrusion of proanthocyanins from Chinese bayberry leaves modifies the physicochemical properties as well as the in vitro digestion of restructured rice. Food Struct..

[67] Kong X., Qiu D., Ye X., Bao J., Sui Z., Fan J., Xiang W. (2015). Physicochemical and crystalline properties of heat–moisture-treated rice starch: Combined effects of moisture and duration of heating. J. Sci. Food Agric..

[68] Englyst H.N., Kingman S.M., Cummings J.H. (1992). Classification and measurement of nutritionally important starch fractions. Eur. J. Clin. Nutr..

[69] Feng Y.Y., Mu T.H., Zhang M., Ma M.M. (2020). Effects of ionic polysaccharides and egg white protein complex formulations on dough rheological properties, structure formation and in vitro starch digestibility of wet sweet potato vermicelli. Int. J. Biol. Macromol..

